# Building better citation practices in public health research: Practical considerations for student researchers, from source discovery to crafting and positioning claims

**DOI:** 10.1371/journal.pgph.0007257

**Published:** 2026-09-09

**Authors:** Ella August, Andrea Mahieu, Andrew F. Brouwer

**Affiliations:** Department of Epidemiology, School of Public Health, University of Michigan, Ann Arbor, Michigan, United States of America; NSCB Government Medical College Department of Surgery: Netaji Subhash Chandra Bose Medical College and Hospital Department of Surgery, INDIA

## Abstract

Discovering, selecting, and citing sources to craft claims is fundamental to public health research and writing. Sources influence our research questions, shape our methodological approaches, connect our work to the broader public health literature, and affect how successfully our writing persuades readers that our research is valuable and that our arguments are sound. Despite the importance of source and citation practices to successful scientific writing, many student researchers receive limited or no formal training on these practices, particularly on drawing from sources to build persuasive arguments. Without specific training, many student researchers struggle to use sources effectively and often encounter pitfalls such as citation bias, quotation errors, and academic plagiarism. In this article, we provide practical considerations for effective source use and citation practices for academic papers. We highlight best practices for source discovery, from strategies for conducting comprehensive literature searches to active reading and synthesis techniques that inform academic thinking and writing. Given the rise of AI, including generative AI and AI-powered research tools, we address both the potential benefits such tools can offer and the potential pitfalls arising from uncritical use. Finally, we discuss selecting and using sources to craft and position claims, including choosing sources to support different types of claims, building credible arguments, and framing citations to reflect a stance or judgment. In this article, we offer practical introductory advice for student researchers to strengthen their source use and citation practices. Using this guidance as a starting point, student researchers can improve the quality of their publications and refine their reading, critical thinking, and writing skills which will serve them throughout their research careers.

## Introduction

The act of discovering, selecting, and citing sources to build persuasive scientific arguments is fundamental to the research and dissemination process in public health and other disciplines. The sources (e.g., scholarly articles, books, reports, data, and websites) that we “discover” (typically through a scholarly database search and reading), select, and cite guide the development and framing of our research questions. They also shape our methodological approaches, connect our work to the broader literature, guide the arguments we build, and impact how successful those arguments are at persuading our readers that our work is meaningful and valuable. Source and citation use is especially important today as trust in public health institutions and policies has declined steadily since the 1970s over a range of metrics [1,2]. Though there are multifaceted and complex reasons for this mistrust [2], the importance of justifying our research and arguments remains critical for our credibility and ultimately the successful administration of public health programs and policies.

In scientific research articles, authors typically make and cite claims in the introduction to highlight the importance of their research, in the methods to justify their methodological approach, and in the discussion to place their findings in the context of the broader field. However, many student researchers in public health receive little guidance on how to approach these aspects of research and writing. Many MPH and public health PhD programs provide limited formal training on source and citation practices and scientific writing. We know of no survey of such training in public health programs, but the limited evidence from adjacent fields suggests a gap in formal training in scientific writing in postgraduate education more broadly [3,4]. Further, while many research and writing guides provide important advice on the structure, style, and formatting of academic articles, including citations, many offer limited or no recommendations about how to find, select, and use sources to support arguments. The guidance that students do receive is often *ad hoc*, usually through one-on-one interaction with their mentors [5]. While a formal study of this gap in training in public health is needed to motivate programmatic change, this essay is intended as an introduction to practical considerations for building better source and citation practices that student researchers can use to improve their scientific writing.

Source and citation practices are inherently complex, shaped by disciplinary norms and principles of effective argumentation [6]. As a team of American quantitative public health researchers, we acknowledge that our perspective and practices may differ from those in other geographic and cultural settings, disciplines, and methodological approaches. Regardless of setting, however, high-quality source and citation practices require proficiency in literature searching, critical reading skills, effective synthesis of information, and the ability to create effective arguments. Good source and citation practices may be complicated by the proliferation of generative AI tools, which are changing the way people both search the literature [7] and write academic papers [8]. Our article offers an introduction to effective source and citation practices for academic papers, ranging from source discovery to their strategic use as in-text citations. Our guidance is informed by the literature from writing studies and library science, as well as perspectives from epidemiology and evidence appraisal in the medical health care literature, among others. We address topics that have been less visible in the published literature, including strategies for selecting citations to support arguments, enhancing the credibility of claims, and strategically positioning them to reflect a stance or judgment about a cited work. This paper fills a gap in practical advice targeted inclusively to early-career public health researchers, including those in low- and middle-income countries. Additionally, our paper includes recommendations about AI tools, which are not included in many existing resources on source and citation use. Additionally, we outline key reading and synthesis strategies to support good citation practices. A main theme in our paper is that high-quality source and citation practices involve rigorous searching, reading, and synthesizing practices. Together, our recommended source and citation practices ultimately strengthen trust and credibility in global health research, particularly as academic research is drawn upon and scrutinized for public reporting and consumption.

## Source discovery: Searching, reading, and synthesizing

### Challenges to source discovery

For student researchers, literature searching and reading are the first steps in the research process. Fully engaging in these activities supports a strong understanding of the state of the field and how your research fits into it. Even for established researchers who are broadly familiar with the literature, regular reengagement and reading widely helps to inform and shape your claims and arguments, and it more effectively positions your work within a broader scholarly conversation. However, many students lack guidance on strategies for searching, synthesizing, and engaging the literature to inform their arguments [3,4]. A resulting pitfall is the use of non-representative citations to support broad claims. Citation bias, for example, includes the overrepresentation of publications presenting statistically significant findings [9], and the highlighting of cherry-picked results. A similar type of pitfall is the exclusion of non-English language articles from review papers [10,11]. An additional challenge is the misrepresentation of findings, referred to as “quotation errors” [12–14]. Misrepresentation can be caused by shallow reading of sources or by relying on secondary sources—including generative AI summaries—that may not accurately reflect the findings of an original study and are more susceptible to misinterpretation [12,15]. The uncritical reliance on AI output can also undermine public health’s credibility more directly. For example, a recent report on children’s health from the US White House included fake, AI-generated citations, which understandably caused readers to question the rigor of the report [16].

### Advice for searching the literature

Employing a comprehensive search strategy will help you capture a representative sampling of the broader literature. Searching multiple databases is helpful, including, for example PubMed, EMBASE, Cochrane Library, Web of Science, Scopus, and Google Scholar. In resource-constrained environments, PubMed Central, SciELO, the DOAJ (Directory of Open-Access Journals), and Research4Life/HINARI are key resources. However, it is important to carefully evaluate the integrity of your sources and take care to avoid predatory publishers [17]. AI-powered tools such as Research Rabbit, used for literature discovery and organization, and Elicit, which summarizes and synthesizes evidence, can be helpful in expanding beyond traditional approaches and making your searches more comprehensive. Though caution should be used with all AI tools, tools like Research Rabbit are less susceptible to the types of pitfalls observed in general-purpose AI tools like ChatGPT—such as hallucinated sources—because they draw from real, indexed databases. However, coverage gaps can be a concern if important literature is not indexed in its sources or if the database is not up to date. Elicit also draws from specific sources, but because generative AI is used to summarize the information, hallucinations and bias are a real risk. To ethically and effectively use these and all AI tools, always verify information against the original source, use the tools as supplements to your own searches, treat the output as a starting point rather than a final product, and understand how the tools work, including the breadth of their coverage, the recency of the information, and whether the tool used generative AI to produce text (as opposed to using AI to select papers or passages without generating text). Please see our AI disclosure for an elaboration of how and where AI was or was not used in this article.

In the early stages of a literature review and appraisal, prioritizing reading review articles and opinion pieces can provide context and big-picture perspectives on your research area, helping you to identify research gaps and refine your research question. Of course, these articles will also supply references that may be a good fit for your paper. Once your research question is finalized, you can focus your review more tightly on your specific question, noting the methodological approaches used, study populations and context, as well as gaps in the literature that can be highlighted in your paper.

As you review the results of your searches, consider the publication dates, article types (e.g., original research, review article, commentary, etc.), as well as the study designs, sample sizes, and study populations. It may be helpful to use search filters to identify only peer-reviewed research, a specific range of publication dates, and other relevant parameters. It is also a good practice to verify whether a citation has been corrected or retracted prior to reviewing it by viewing the article on the publisher’s website. However, we recommend avoiding filtering out studies by language as this can result in a form of citation bias [10,11]. In fact, linguistic bias marginalizes researchers from non-English-speaking regions, contributing to disparities across the scientific community [18–21]. Articles published in different languages can be translated with one of many free electronic translators available online (e.g., Google translate). Identifying local literature through sources such as African Index Medicus, LILACS, or other databases can be helpful to be more inclusive of local perspectives and data.

Searching the literature should be an ongoing process; given that manuscript publication can take up to two years or more from when you start drafting, regularly revisiting your searches will help you to capture new papers as they are published and integrate new studies into your writing. Organizing your sources into a free citation manager such as Zotero or Mendeley can help you keep track of your references over time, even those that do not end up in your final reference list.

### Advice for reading and synthesizing the literature

As you search the literature to identify articles of interest and move toward a more careful evaluation of a subset of articles, you will need to employ specific reading strategies. Whereas skimming is helpful in weeding out irrelevant articles, close reading is important when evaluating the methodological rigor and validity of findings and helps you accurately represent the source material in your writing [22]. Reading the articles also inspires new ideas and insights and helps you understand how your study findings fit into the broader literature. Importantly, part of the source discovery and reading process is taking note of multiple, potentially synergizing or conflicting, sources. This synthesis underscores the importance of treating reading as a thinking process, and it will set you up for success when you start building claims.

Reading and writing are iterative processes, and remaining open as you read will allow you to embrace evidence that contradicts your current thinking, providing the opportunity to revisit and fine tune your arguments. This process can be trying; Kamler [5] describes the revision process as analogous to unstitching “a garment that took so long to design.” Nevertheless, maintaining an open mind and flexible approach fosters strong writing and rigorous scientific inquiry.

Active reading helps you more deeply engage with and synthesize information from sources [22]. There are many approaches to reading actively, but one particularly helpful method is the creation of text annotations [22]. Broadly, text annotations involve actively responding to and creating meaning from a text to help you comprehend the information and generate new ideas [23]. One way to annotate a reading is to make notes, adding comments and questions about the source; these notes serve as a bridge to launch an initial draft of your paper. Though generative AI tools can be helpful for academic research and writing, keep in mind that they are not substitutes for reading and evaluating sources oneself and that generative AI is not a primary source [24].

## Selecting and using sources to craft and position claims

### Challenges to using sources to craft and position claims

Once you have read enough to position your paper in the broader literature, the process of drafting your text and crafting claims begins. In some cases, crafting a specific claim may prompt you to revisit the literature to identify an additional source to support an assertion; the process is rarely linear.

Students can be unsure of which types of studies are best suited to certain types of claims, as well as the appropriateness of citing one’s own research. Additionally, approaches to positioning claims—i.e., choosing how you describe a study and its results to convey your opinion of its importance, credibility, and relevance—are often not discussed in training programs. Finally, crafting strong arguments can also be a challenge, particularly for student researchers. Some may be eager—but uncertain—about how to choose the most effective sources for their purposes. One prevalent pitfall in this area is academic plagiarism, which includes the misrepresentation of someone else’s idea or language as one’s own [25]. By one estimate, 30% of manuscripts submitted to a journal were judged to include plagiarized text (plagiarism in this case was broadly defined, including self-plagiarism) [26]. In the next section, we offer advice for selecting sources and crafting and positioning claims, highlighting specific advice for different sections of the paper [27,28].

### Typical claims in the introduction, discussion, and methods sections

Different types of claims tend to be made in the three manuscript sections that feature citations: the Introduction and Discussion sections, which can have similar citation goals, and the Methods section, which has different needs for citations.

In the Introduction, authors typically support claims about the importance of the problem the research addresses, often focusing on how widespread a public health problem is and the associated morbidity and mortality. These arguments justify the need for your study. Claims about prior work are also included, such as those describing studies that led to your research, as well as arguments about the limitations or gaps in existing work that the current study seeks to fill. Your claims and citations in the Introduction should be specific to the problem you seek to address and your research question. To that end, we recommend avoiding “primacy claims” that justify the need for your research by claiming that your team is the first to study it. That others have not evaluated a particular question (or that little research exists) is not an adequate justification for conducting a research study. Instead, state the need for your research by describing the outcome that could result from addressing the problem with your study (e.g., reduced malaria prevalence through improved spatial targeting of mosquito control) [29].

The Discussion section provides context for readers to understand your results. Important functions of citations in this section include helping readers compare your study to similar research, placing your investigation into the broader field, and highlighting new insights. Many of the same approaches work here as in the Introduction. However, in the Discussion, the choice of which sources to include may depend less on their quality and rigor and more on the relevance of the topic; selected sources should facilitate direct comparison with the current study and highlight novel insights and outcomes of your research. You should compare and contrast your findings with the results of other studies, choosing sources that both align and do not align with your results. Citing sources that are consistent with your results can strengthen your arguments by providing additional support for your specific finding or suggesting greater generalizability; however, eliding over sources that are not consistent may lead readers to see you as “cherry-picking,” reducing your credibility. Additionally, including sources with differing results or claims enables you to highlight insights and implications of your research. You may also decide to reference select sources that diverge from the broader literature if they offer important implications for interpreting your results.

In the Methods, your claims and citations should describe how you carried out your study and justify why you chose the methods employed. This section should enable readers to interpret your results in light of how they were generated. The content and citations selected will depend on the study design and type, but they likely include justifying the instrument selection and supporting an analytic approach. Consult a relevant reporting guideline specific to your study design, such as one in the EQUATOR Network, to guide your methods content, which will shape your citation approach. In the case of an ongoing longitudinal study or nested study, citing earlier design protocol papers or results papers from the main study can provide helpful context and save words. The Methods can be strengthened by citing a landmark or seminal paper describing a well-known method for recognition or a very recent source for a more cutting-edge method. If your approaches differ from cited practice, then describe how. If you used existing protocols or survey instruments (including one you previously published), cite them in the Methods, as well. These approaches will improve transparency, which is critical in gaining the trust of readers.

### Advice for selecting sources to build and support claims

Across these three manuscript sections, *broad statements* (e.g., “tuberculosis is an entrenched health issue worldwide”, “there are concerns that e-cigarettes may lead youth to initiate cigarette smoking”) may be best supported by more expansive research approaches, such as umbrella or systematic reviews. While some authors have discouraged citing review papers, characterizing them as “secondary sources,” and thus an indirect link to a claim [28], we advocate for their use in this context because they are better positioned to support the state of evidence on a topic compared to a single study. In fact, evidence hierarchies like the GRADE approach advocate prioritizing systematic reviews and meta-analyses for these purposes, as they are designed to provide guidance for rating the quality of evidence and the strength of recommendations in health care [30].

When selecting sources to support *specific claims* (e.g., “tuberculosis kills more people globally than any other disease caused by an infectious agent”, “in the US, 5% of adults currently vape e-cigarettes”), choose studies with stronger study designs. Although randomized controlled trials are considered the gold standard for supporting causal associations, these studies are often not feasible in or translatable to public health contexts. Other strong study designs common in public health include longitudinal cohort and case-control studies [31]. In contrast, results from cross-sectional and ecologic studies provide weaker evidence for causal inference [31]. Consistent with this perspective, citing abstracts (e.g., conference abstracts) should be avoided if possible, particularly in cases where they were not peer-reviewed and do not contain complete information about a study [24], as they limit the reader’s ability to judge the quality of the study. Beyond these general principles, you should be aware of sub-discipline-specific evidence paradigms, such as the GRADE-CERQual framework for qualitative research [32], the use of realist synthesis in implementation science [33] or the UK Medical Research Council’s framework for complex interventions [34].

The reputation of both the journal and authors cited can also lend credibility to your arguments and foster trust in your overall product. Citing a well-known and respected institutional author like World Health Organization or a publication in a highly visible journal such as Lancet Public Health can help to strengthen a claim. However, be careful of the citation bias of selecting references just because they have many previous citations. Additionally, some reviewers will look to see if you have cited papers published in the journal you are submitting to, demonstrating that your work is relevant to the specific readership.

Additional considerations for assessing the quality and relevance of a potential citation include the methodological rigor of the study, the representativeness of the findings with respect to the broader literature, sample size, and recency of the publication date. Another consideration is whether the scope of the research study is appropriate to support a claim. For example, the paper “Accuracy of citation and quotation in foot and ankle surgery journals” published in *Foot and Ankle International* may not be suitable for supporting a claim about the prevalence of citation inaccuracy across the health sciences more broadly. Likewise, a small study done in a specific geographic region or subpopulation may not be appropriate for a more general claim about a given research finding. (For example, within our own Introduction, we needed to be careful that our citation of Greenwood et al. [4], one of the few existing studies of scientific writing training, which surveyed 244 nursing students, is presented as weak, adjacent evidence and not definitive evidence of a lack of scientific writing training in public health). If the goal is a generalization, it is typically appropriate to cite multiple related papers to support a single claim. Or, if the claim is specific to a given population, then the source should be relevant to that specific population.

### Advice for positioning your claims and citations

It is not possible to be completely neutral when citing others’ work, nor should it be your goal [35,36]. Instead, make deliberate choices about how you frame the studies you cite, being intentional in your word choice when introducing a reference. As Penders notes [37], citing transparently provides context for arguments that allows readers to understand the relevance of a reference, the rationale for its inclusion, and the authors’ stance towards it [35,37]. (For example, in the previous sentence, the phrase “As Penders notes” positions Penders as an authority on the topic and suggests that the forthcoming remark will be a straightforward agreement with or addition to our argument.) In cases where evidence on a topic is equivocal or there are a range of viewpoints, be transparent in describing and citing different perspectives and evidence, including work that does not directly support your own ideas or findings. Reasons for citing a source vary, and you will not necessarily endorse or agree with all the citations you discuss. Choose your language carefully when describing a source or study, signaling to readers your stance toward it. Penders [37] and Lingard [35] advocate for positioning your citations with this approach, offering suggestions for particular verbs that can help us provide this additional context. Interpreting study results with the word “suggests,” for example, is more tentative than the phrase “provides compelling evidence.” Though we currently know of no systematic, discipline-specific taxonomy of citation verbs organized by the degree of epistemic commitment—i.e., the strength of assertion that a statement is true—the development of such a resource would make a meaningful contribution to this area and help researchers consider and communicate their claims more precisely.

When referring to source material, use your own words to paraphrase information, reserving direct quotations for the instances where the exact wording is needed for context or clarity (rare in scientific writing). For example, when describing the specific instructions provided to participants in a hand-washing intervention or the specific wording of a sensitive item on a questionnaire, it may be necessary to quote the exact language used. In most circumstances, however, it is your role to interpret, synthesize, and paraphrase information from sources to create your own arguments and analysis. On a more practical note, to be clear about which citation supports which claim, we recommend inserting relevant citations immediately after each claim, even if it is mid-sentence [38].

Finally, while some authors endorse avoiding self-citations [39], others embrace them as an essential part of scientific communication. Mishra et al. [40], for example, refer to self-citation as “the hallmark of highly productive authors.” Our advice, however, aligns with those who advocate for a balanced approach, suggesting that self-citation is appropriate in certain contexts [28,37,41], such as when building on your previous work, when it provides related background for your study or methodological approach, or in comparing your findings to other research. However, self-citation becomes problematic when it is used to promote self-interest or when it leads to citation bias or presents an unbalanced or unrepresentative view of the literature. However, despite a plethora of opinions and advice as well as numerous reports featuring self-citation counts and their impact on citation metrics, there is a lack of literature in the health sciences rigorously classifying different types of claims supported by self-citation in published articles and reflection upon the appropriateness of different uses. This type of analysis would provide important insights and improve the clarity and specificity of advice in this realm.

## Conclusion

Source discovery, selection, and the positioning of your claims play a central role in scientific writing in public health, affecting how you think about and frame your work and how persuasive and trustworthy your readers find your arguments. The reading, thinking, and writing processes are intrinsically intertwined, and nurturing each will improve the others.

Key strategies for high-quality source and citation use to craft persuasive arguments and overall credibility build on a comprehensive search strategy that is inclusive of different languages and local literature where relevant. Also important is the quality and relevance of the source and research, considering the recency of the publication and the reputation of the authors and journal. Choose a study that supports the type of claim you are making: consider the topic area, population, study design, data, strength of evidence, and section of your paper when crafting a claim. Position your arguments to signal your own stance toward a citation but acknowledge opposing viewpoints if there are important ones. AI tools, for better or worse, are now part of the academic and writing landscape, and they can be helpful if used critically and transparently, staying engaged throughout their use.

While this essay is not a comprehensive review paper, providing neither a framework nor an exhaustive discussion of pedagogical approaches supporting good source and citation practices, our hope is that it provides student researchers with practical guidance on source and citation best practices that can improve their scientific writing. Additionally, we hope that this paper will inspire future discussion and debate leading to the inclusion of more structured resources, guidance, and education on source and citation use globally within MPH and public health doctoral programs. Training approaches will necessarily vary across geographic regions, cultures, and disciplines as the values, resources, educational traditions, and contexts differ considerably.

As highlighted earlier, future research should systematically assess what training educational programs are currently offering in this area and the level of competency students achieve. We recommend developing supporting resources, such as a systematic, discipline-specific taxonomy of citation verbs, data-driven insights on self-citation practices, and other resources suggested in this article which would be in the service of helping researchers consider and communicate their claims more precisely.
